# Sugar Coated Pills

**Published:** 1875-05

**Authors:** 


### Sugar Coated Pills.

At a late meeting of the Pharmacentical Society of Philadelphia, Dr. Miller spoke strongly against the purchase of cheap sugar coated quinia pills. There in the market forty-five thousand such pills which do not contain a trace of quinia. They were made from muriate of cinchonia, furnished by a New York house as sulphate of quinia to some of the makers of sugar coated pills.—*New York Evening Post*, *May* 14.

When the plan of sugar coating pills was first brought to the notice of the profession it was very generally commended, and the Hieroglyphics S. C. became a uaual addition to every prescription in which medicines were prescribed in the pilular form. The elegant appearance of these pills with their glossy saccharine coating so effectually concealing the taste and even smell of nauseous and repulsive remedies seemed to leave nothing to be desired, and their popularity was such that the plan was adopted by nostrum venders and Homeopathists, and almost every variety of drug from Cod Liver Oil to Dovers Powder was offered in this form to the regular profession, and readily swallowed by the public.

The preparation of sugar coated pills soon became a special branch of Pharmacy, and large fortunes were embarked in the business.

The usual competition in prices soon resulted, and the business which had at the first yielded large profits was done on very small margins.

Unprincipled parties were not slow to discover, however that to increase profits and reduce prices it was only necessary to use cheap drugs, or to substitute for the more expensive chemicals, those of a similar nature but cheaper, while the finished pellet with its white and glossy covering concealed all defects and *sold well* to these sharp buyers who are always on the lookout for *bargains.* The consequence has been that the market has been flooded with sugar coated pills which, as many physicians have found to their sorrow, have either been almost inert, or as in the case of cathartics have operated with a severity which is easily accounted for, if for a prescription for pil. coloc. comp. the unsuspecting patient has received a compound of cape aloes and croton oil; or if, as noted in the extract from a New York paper, with which we have prefaced these remarks, pills of cinchonine are labled sulphate of quinine and dispensed as such to the malaria stricken patient, is it surprising that we frequently do not get the results expected from sugar coated quinine pills. Again, it is the custom of some makers to mix the sugar used in the coating of pills with a large proportion of Terra Alba, and in many cases even where there is no fraudulent substitution or inert drugs, the pills are in this preparation subjected to so great a heat that they become hard as adamant, and absolutely insoluble in the alimentary canal.

From such experiences many physicians have entirely discarded the use of sugar coated pills, but at the same time it is undeniable that if *reliable* and soluble pills can be obtained, the sugar covered form, is for very many nauseous medicine decidedly preferable to any other.

We fully believe that all of these essentials are fulfilled in the pills prepared by some of the reliable manufacturers, and physicians in order to obtain the desired results in prescribing medicines in this form should see to it that their prescriptions are dispensed by druggists wholly above suspicion

